# The Impact of Genome Region of Difference 4 (RD4) on Mycobacterial Virulence and BCG Efficacy

**DOI:** 10.3389/fcimb.2017.00239

**Published:** 2017-06-08

**Authors:** Huanwei Ru, Xiaojia Liu, Chen Lin, Jingyan Yang, Fuzeng Chen, Ruifeng Sun, Lu Zhang, Jun Liu

**Affiliations:** ^1^State Key Laboratory of Genetic Engineering, School of Life Science, Institute of Genetics, Fudan UniversityShanghai, China; ^2^Key Laboratory of Medical Molecular Virology of Ministries of Education and Health, Fudan UniversityShanghai, China; ^3^Shanghai Engineering Research Center of Industrial MicroorganismsShanghai, China; ^4^Department of Molecular Genetics, University of TorontoToronto, ON, Canada

**Keywords:** RD4, *Mycobacterium tuberculosis*, genome, bacterial, *Mycobacterium marinum*, *Mycobacterium bovis* BCG

## Abstract

Comparative genome analyses have revealed a number of regions of difference (RD) among mycobacterial species. The functional consequences of most of these genome variations have not been studied. RD4, which encompasses Rv1506c-Rv1516c of *Mycobacterium tuberculosis* (*M. tb*) H37Rv, is absent in the closely related *Mycobacterium bovis* and *M. bovis* Bacille Calmette-Guérin (BCG). On the other hand, we previously found that *Mycobacterium marinum* has an extended RD4 which includes a number of genes involved in the biosynthesis of lipooligosaccharides (LOSs). As such, there appears to be a gradual decay of RD4 in mycobacterial genomes in the order of *M. marinum, M. tb*, and *M. bovis* (including BCG). To understand the potential effect of RD4 on mycobacterial virulence, in this study, we cloned the entire (Rv1501–1516c) and partial (Rv1501–1508c) RD4 into an integrating vector. These constructs were introduced to *M. bovis* BCG and *M. marinum* and the virulence of the RD4 knock-in strains were evaluated in the SCID mice and zebrafish infection models, respectively. BCG containing the entire RD4 exhibited similar levels of virulence to the parental strain but BCG containing partial RD4 (Rv1501–Rv1508c) was more attenuated. Similarly, zebrafish infection experiments showed that addition of partial RD4 also appeared to attenuate the virulence of *M. marinum*. However, *M. marinum* containing entire RD4 was more virulent than the wild type strain. Interestingly, BCG strains containing the entire or partial RD4 exhibited better protection of zebrafish against *M. marinum* challenge than the parental BCG. Taken together, our data suggest that RD4 plays a role in mycobacterial virulence and that RD4 knock-in BCG strains confer improved protection. Our study has provided new insights into the biological function of RD4 and evolution of mycobacterial genomes.

## Introduction

The *Mycobacterium tuberculosis* (*M. tb*) complex (MTBC) comprises a group of closely related subspecies that shares 99.9% identical genome sequences but differs widely in terms of host tropisms and pathogenicity. These include the human-adapted *M. tb, Mycobacterium africanum*, and *Mycobacterium canettii* and the animal-adapted *M. bovis, Mycobacterium caprae, Mycobacterium microti, Mycobacterium pinnipedii, Mycobacterium orygis*, and *Mycobacterium mungi* (Brosch et al., 2002; Comas et al., 2013). Bacille Calmette-Guérin (BCG) is derived from *M. bovis* and is currently the only approved tuberculosis (TB) vaccine (Liu et al., 2009). Comparative genome analyses have identified at least 20 variable regions among the genomes of the MTBC, which are results of insertion-deletion events. For examples, compared to *M. tb* H37Rv, 14 regions of difference (RD1-14), ranging in size from 2 to 12.7 kb, are absent in the genome of *M. bovis* BCG-Pasteur (Mahairas et al., 1996; Brosch et al., 1998, 2002; Behr et al., 1999; Gordon et al., 1999; Mostowy et al., 2003). Conversely, six regions (RvD1-6) are absent in the genome of *M. tb* H37Rv but are present in *M. bovis* (Mahairas et al., 1996; Brosch et al., 1999; Gordon et al., 1999). Analyses of the distribution of these variable genome regions in multiple MTBC strains isolated from different human populations and diverse animal species have established the genetic lineage of the MTBC (Brosch et al., 2002).

Presumably RD regions are involved in host interactions and pathogenicity, which determines the host spectrum of individual species of the MTBC. Consistent with this notion, RD1, which is absent in all BCG vaccine strains but present in virulent *M. tb* and *M. bovis*, encodes a virulence-associated type VII secretion system (Mahairas et al., 1996; Behr et al., 1999; Abdallah et al., 2007). This system includes two secreted proteins ESAT-6 and CFP-10, which are important for virulence, and a secretory apparatus encoded by the surrounding genes in RD1 (Hsu et al., 2003; Stanley et al., 2003). RD2, which is absent in a subset of BCG strains (BCG strains distributed after 1927), was also found to be involved in virulence (Kozak and Behr, 2011). The distribution of other RD regions among *M. tb* and *M. bovis* strains is not uniform and their functions are less well-characterized (Brosch et al., 2002).

RD4, a 12.6 kb fragment that contains 11 genes (Rv1506c–1516c) of *M. tb* H37Rv and appears to be involved in the biosynthesis of trehalose containing glycolipids (Brodin et al., 2010), is absent in the most common *M. bovis* strains including *M. bovis* BCG (Mahairas et al., 1996; Brosch et al., 1998, 2002; Behr et al., 1999; Gordon et al., 1999; Mostowy et al., 2003). Interestingly, we previously found that *M. marinum*, a fish pathogen that is closely related to the MTBC, contains an extended RD4 region which includes >40 genes that are involved in the biosynthesis of lipooligosaccharides (LOSs) (Ren et al., 2007). However, *M. tb* cannot produce LOSs likely due to the recombination of two *pks5* genes and deletion of the intergenic *pap* gene, which in the closely related progenitor species *M. canettii* are situated next to the RD4 locus (Boritsch et al., 2016). Therefore, there appears to be a gradual decay of RD4 in the mycobacterial genomes, which is in the order of *M. marinum, M. tb*, and *M. bovis* including BCG. To test our hypothesis that RD4 could play a role in mycobacterial virulence, we constructed RD4 knock-in strains of *M. bovis* BCG and examined their virulence and protective efficacy. We also examined whether expression of the RD4 in *M. marinum* affects its virulence.

## Materials and methods

### Bacterial strains and culture conditions

*Mycobacterium bovis* BCG strains were grown at 37°C in Middlebrook 7H9 broth (Difco™) supplemented with 0.2% glycerol, 10% albumin-dextrose-catalase (ADC; BD BBL™), and 0.05% Tween 80 or on Middlebrook 7H11 agar (Difco™) supplemented with 0.5% glycerol and 10% oleic acid-albumin-dextrose-catalase (OADC; BD BBL™). *Mycobacterium marinum* strains were grown in the same media except the growth temperature was at 30°C. *Escherichia coli* strain DH5α was used for routine manipulation and propagation of plasmid DNA. *E. coli* strains were grown in LB broth or agar. Antibiotics were added as required: kanamycin, 50 μg/mL for *E. coli* and 25 μg/mL for BCG or *M. marinum* strains.

### Molecular cloning

The integrating vector pMV306 (Stover et al., 1991) was used for the cloning. We first constructed pMV306::Rv1501-1502. The forward primer 5′-CACTGGTCGACAATGTCACTTCATTTAGCAAC-3′ and reverse primer 5′-CATGAAAGCTTCGAATCATTGGAACAGCGG-3′ were used for PCR amplification using *M. tb* genomic DNA as template. The PCR fragment was digested with SalI and HindIII and ligated into pMV306 predigested with these two enzymes. To construct pMV306::Rv1501-1508c, the forward primer 5′- CCTCGAAGCTTTCATGATACCGGTTCCATAGGTCCAATC-3′ and reverse primer 5′- TTGGCTAGCAACCGCGCGAGGTCCTC-3′ were used for PCR amplification using *M. tb* genomic DNA as template. The PCR fragment was digested with HindIII and NheI and ligated into pMV306::Rv1501-1502 predigested with HindIII and XbaI. To construct pMV306::Rv1501-1516c, the bacterial artificial chromosome Rv264 (Brosch et al., 1998) was isolated and digested with HindIII, NheI, and BglII. A10-kb fragment from the digestion mixture was gel purified and ligated to pMV306::Rv1501-1502 predigested with HindIII and XbaI. Standard electroporation protocols were used for transformation of *M. bovis* BCG and *M. marinum* with pMV306::Rv1501-1508c or pMV306::Rv1501-1516c. Transformants were selected on Middlebrook 7H11 agar containing kanamycin (25 μg/mL).

### Reverse transcription and polymerase chain reaction (RT-PCR) analysis

Mycobacterial cultures (5 mL, OD_600_ = 1.0) were pelleted and resuspended in 800 μL Trizol. Cells were disrupted by bead beating. The supernatant was then extracted with chloroform:isoamyl alcohol (24:1) and precipitated with isopropanol. Crude RNA samples were treated with gDNA Eraser and reversely transcribed using the PrimeScript™RT reagent Kit (Takara) according to the manufacturer's protocol. The resulting cDNA was used as the template for PCR amplification using primers specific to Rv1501 (5′-GGCGCTAGCATGATTCCTGTAAAGGTTGAAAACAATAC-3′, 5′-TTTCAAGAAAGGTAAAGAAATGAGGGTCATAC-3′), Rv1507c (5′-TGTGCTAGCTTGAAGAAAGTCGCGATTGTTCAATC-3′, 5′-CGTGTGCTGTTCTTCGAGGTAAATCGGCGCG-3′), and Rv1516c (5′-TATAAGCTTTCCGAATCCCTTGTGAAGTAGTAATGTGCGAGC-3′, 5′-CGATCCAGTAGTCGTCCGCCTCGCACAACGC-3′).

### Antisera preparation and western blot analysis

The open reading frame of Rv1505c was amplified by PCR using the primers 5′-GCGGATCCATGACCAAACCATTGGTAATT-3′ and 5′-GTAAGCTTTCATATCTTCCGCAACTCCGTGC-3′. The PCR product was digested with BamHI and HindIII and cloned into pET28a (Novagen), and *E. coli* BL21 was transformed with the resulting expression construct. To overexpress the Rv1505c protein, *E. coli* BL21/pET28-Rv1505c culture was grown at 37°C for 3 h and before being induced with 1 mM IPTG for 4 h. Rv1505c protein was purified from the inclusion body. Briefly, the cultures collected after IPTG induction were resuspended with PBS and sonicated to break cells. The cell lysate was centrifuged at 12,000 rpm for 20 min at 4°C. The resulting pellet was resuspended with 20 mM Tris, pH 8.0, 100 mM NaCl, and 8 M urea, and was then subjected to Ni-NTA His•Bind® Resin. Purified protein was centrifuged using microfuge tubes with a low molecular weight cut off filter to remove urea and imidazole.

Four C57BL/6 mice were immunized subcutaneously with a mixture containing 100 μl purified Rv1505c protein (10 μg) and 100 μl of Freund's incomplete adjuvant (Sigma). The immunization procedure was repeated two more times (2 weeks apart). Two weeks after the last immunization, mice were sacrificed and their sera were collected.

For analysis of Rv1505c expression in recombinant BCG and *M. marinum* strains, cell lysates were prepared and subjected to standard Western blot analysis. The primary antibody was the antisera against Rv1505c protein prepared above (1:100 dilution).

### Macrophage infection

Infection of murine macrophage cell line J774A.1 with recombinant strains of *M. bovis* BCG-China and *M. marinum* 535 was performed as previously described (Ramakrishnan and Falkow, 1994; Tan et al., 2006; Ren et al., 2007). Briefly, J774 cells were maintained at 37°C in 5% CO_2_ in Dulbecco's Modified Eagle medium (DMEM) (Gibco) supplemented with 10% fetal bovine serum (FBS) (Gibco). Cells were seeded at a density of 10^6^ cells per well in DMEM medium supplemented with 10% FBS for 16 h. Macrophage cells were then infected with BCG or *M. marinum* at multiplicity of infection (MOI) of 10 and 1, respectively, for 4 h. Cells were washed three times with PBS and subsequently, incubated at 37°C (for BCG) or 32°C (for *M. marinum*) in fresh media containing 20 μg/mL gentamycin. At various time points post-infection, the infected macrophage monolayers (three wells per strain) were washed twice with fresh media and then lysed with 0.1 ml of 1% Triton X-100 (Sigma) to release intracellular mycobacteria. The number of intracellular mycobacteria was enumerated by plating appropriate dilutions on Middlebrook 7H10 agar plates containing appropriate antibiotics.

### Ethics statement

All of the animal procedures were approved by the local animal care committees at Fudan University. All methods were performed in accordance with the relevant guidelines and regulations.

### Analysis of BCG virulence in SCID mice

SCID mice infection with BCG was performed as described previously (Zhang et al., 2016). Briefly, age-matched (6 weeks) female SCID mice were purchased from Beijing HFK Bioscience Co. Mice (22 per group) were infected intravenously via the tail vein with 10^7^ CFU of the different BCG strains in 0.1 ml PBS/0.01% Tween-80 and were monitored for survival. At day 1 post-infection, 2 mice from each group were sacrificed and the lungs and spleens were harvested, homogenized in PBS, and plated on 7H11 agar to enumerate bacterial burden. This was performed to confirm the actual infection dosage: 5.92 ± 0.10 log_10_, 6.16 ± 0.14 log_10_, and 6.15 ± 0.09 log_10_ CFUs in the spleens and 4.43 ± 0.11, 4.42 ± 0.10 log_10_, and 4.45 ± 0.12 log_10_ CFUs in the lungs for BCG-China::pMV306, BCG-China::Rv1501-1508c, and BCG-China::Rv1501-1516c groups, respectively. There was no significant difference on the infection dosage among the three groups.

### Analysis of *M. marinum* virulence in zebrafish infection

Zebrafish infection with the recombinant strains of *M. marinum* was performed as previously described (Dong et al., 2012). Briefly, adult zebrafish (26 per group) were infected by intraperitoneal injection with a dosage of 100 CFU bacteria per fish or PBS as the negative control and monitored for their survivals.

### Analysis of protective efficacy of recombinant BCG in zebrafish-*M. marinum* infection model

Groups of adult zebrafish (20 per group) were vaccinated intraperitoneally with 10^4^ CFU of recombinant BCG or PBS as the negative control. Thirty days after BCG vaccination, zebrafish were infected intraperitoneally with 10 CFU of *M. marinum* 535 and were monitored for survival. In another experiment, Groups of adult zebrafish (15 per group) were vaccinated intraperitoneally with 10^4^ CFU of recombinant BCG or PBS as the negative control. At day 30 post-infection, six live fish from each group were sacrificed and homogenized. The homogenates were plated by serial dilution on Middlebrook 7H11 agar and incubated at 30°C for 7 days to determine the CFU of *M. marinum*.

## Results

### Generation of RD4 knock-in strains

**Figure 1:**
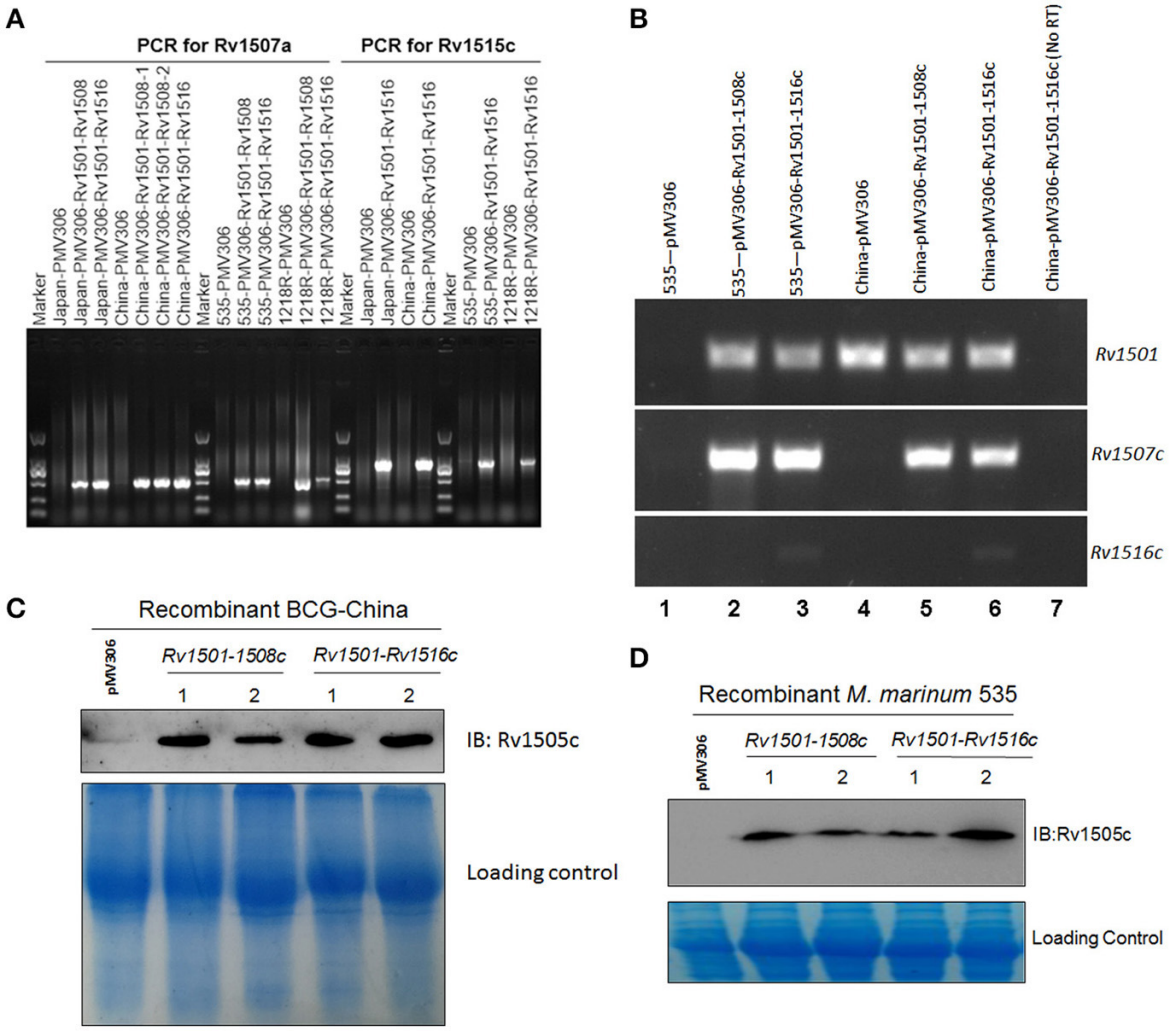
Molecular characterizations of RD4 knock-in strains of *M. bovis* BCG and *M*. *marinum*. **(A)** PCR analysis of mycobacterial genomic DNA. Chromosomal DNA from two BCG strains (BCG-Japan, BCG-China) and two *M. marinum* strains (1218R, 535) transformed with pMV306-Rv1501-1508c or pMV306-Rv1501-1516c were isolated and used as the template for PCR amplifications. PCR primers specific for Rv1507a and Rv1515c were used to amplify these two genes. Rv1507a was detected in all strains harboring Rv1501-1508c or Rv1501-1516c. Rv1515c was detected in strains harboring Rv1501-1516c. Strains transformed with empty vector pMV306 were used as the negative control. **(B)** RT-PCR analysis of the expression of Rv1501, Rv1507c, and Rv1516c in recombinant strains of BCG-China and *M. marinum* 535. Lanes 1-6: RNA was isolated from indicated strains and treated with DNase, which was then subjected to reverse transcription PCR analysis; lane 7: the same sample as lane 6 except no RT-PCR was performed. **(C,D)** Western blot analysis using antisera against Rv1505c. Cell lysates were prepared from indicated strains and subjected to Western blot analysis. The low panel in each figure is the Coomassie blue staining which served as the loading control.

We successfully cloned two fragments, which cover partial (Rv1501-1508c) and entire (Rv1501-1516c) regions of RD4, respectively, into a mycobacterial integrating vector pMV306. These constructs were introduced to two *M. bovis* BCG strains (BCG-China and BCG-Japan) and two *M. marinum* strains (535 and 1218R). PCR analysis using primers specific to Rv1507A (for Rv1501-1508c) and Rv1515c (for Rv1501-1516c) confirmed the successful integration of these two fragments into the mycobacterial genomes (Figure 1A). To determine if genes in these two fragments are expressed, we performed RT-PCR analysis and examined the expression of Rv1501, Rv1507c, and Rv1516c in representative organisms (Figure 1B). Results showed that Rv1501 and Rv1507c transcripts were detected in all knock-in strains, while Rv1516c was expressed only in the strains containing the entire RD4. This was further confirmed by Western blot analysis using antisera against Rv1505c (Figures 1C,D).

**Figure 2:**
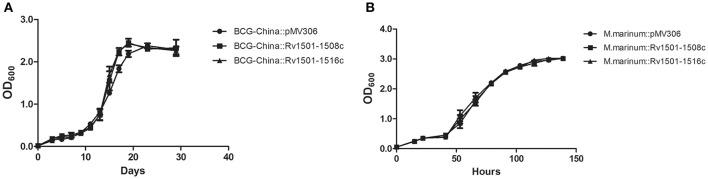
*In vitro* growth of RD4 knock-in strains of *M. bovis* BCG and *M. marinum*. Recombinant strains of BCG-China and *M. marinum* 535 were grown in 7H9 broth at 37°C **(A)** and 30°C **(B)**, respectively. Data are from triplicate samples of each strain (mean ± *S.D*.).

**Figure 3:**
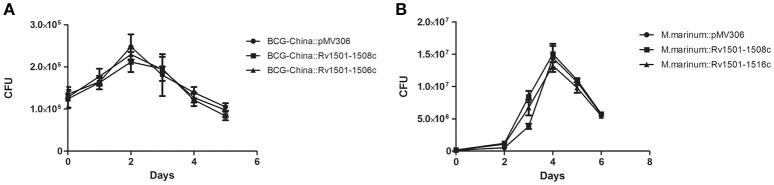
Multiplication of RD4 knock-in strains of *M. bovis* BCG and *M. marinum* in macrophages. J774 macrophages were infected with recombinant strains of BCG-China (MOI = 10) **(A)** or recombinant strains of *M. marinum* 535 **(B)** (MOI = 1). At various time points, the intracellular bacterial number was determined and plotted. Data are from triplicate samples of each strain (mean ± *S.D*.).

Next, we used BCG-China and *M. marinum* 535 knock-in strains as the representatives and examined their growth in 7H9 broth and in J774 macrophages. There was no difference in growth under these conditions between the knock-in strains and the respective control strains (Figures 2, 3).

### Virulence of knock-in strains

We first determined the virulence of the BCG-China knock-in strains in SCID mice. Since BCG is highly attenuated, the virulence of BCG strains is routinely evaluated in immunocompromised SCID mice lacking T- and B-lymphocytes. Groups of SCID mice (*n* = 20) were intravenously infected with 10^7^ CFU of the BCG strains and their survivals were monitored. The survival curves were plotted using a Kaplan-Meier analysis.

**Figure 4:**
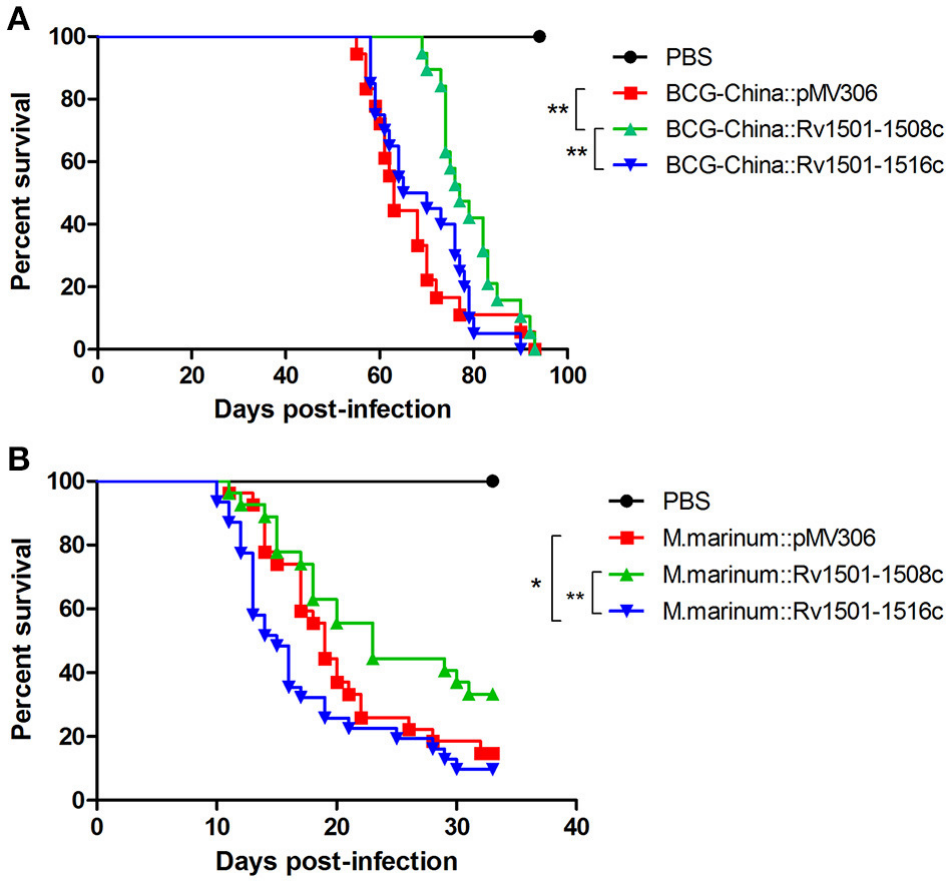
Virulence of RD4 knock-in strains of *M. bovis* BCG and *M. marinum*. **(A)** Survival curves of SCID mice infected with recombinant BCG-China. Groups of SCID mice (*n* = 20) were intravenously infected with 10^7^ CFU of BCG strains and their survivals were monitored. **(B)** Survival curves of zebrafish infected with recombinant *M. marinum* 535. Groups of adult zebrafish (*n* = 26) were intraperitoneally infected with 100 CFU of *M. marinum* strains and monitored for survival. Log-rank test (Mantel-Cox) was performed for statistical significance. ^*^p < 0.05; ^**^p < 0.01.

The median survival time for the parental, the BCG-China::Rv1501-1508c and BCG-China::Rv1501-1516c groups were 63, 77, and 67.5 days, respectively (Figure 4A). Log-rank analysis revealed that the BCG-China::Rv1501-1508c group survived significantly longer than the parental BCG group (*p* < 0.01) and the BCG-China::Rv1501-1516c group (*p* < 0.01). There was no significant difference between the parental and the BCG-China::Rv1501-1516c groups.

We next determined the virulence of *M. marinum* 535 knock-in strains in zebrafish. Zebrafish have been widely used as a laboratory model for studying *M. marinum* infection, which manifests both acute disseminated disease and chronic persistent infection (Tobin and Ramakrishnan, 2008). Groups of adult zebrafish (*n* = 26) were intraperitoneally infected with 100 CFU of WT *M. marinum* 535 and the two RD4 knock-in strains, and were monitored for survival. The median survival time for the WT, *M. marinum*:: Rv1501-1508c, and *M. marinum*::Rv1501-1516c were 19, 23, and 15 days, respectively (Figure 4B). Zebrafish infected with *M. marinum*::Rv1501-1516c succumbed to disease significantly sooner than that infected with the *M. marinum*::Rv1501-1508c (*p* < 0.01) and the WT *M. marinum* (*p* < 0.05). The *M. marinum*::Rv1501-1508c group appeared to survive longer than the WT group with the difference approaching significance (*p* = 0.08).

### Protective efficacy of BCG knock-in strains

Since the RD4 region may contain important antigens that are missing in BCG, we also examined if the RD4 knock-in strains of BCG exhibited better protection against mycobacterial challenge using the zebrafish-*M. marinum* infection model. Oksanen et al. previously showed that vaccination of with BCG or DNA vaccines expressing known antigens (Ag85B, ESAT-6 and CFP-10) protects adult zebrafish against *M. marinum* infection, suggesting that zebrafish is a promising new model for preclinical TB vaccine research (Oksanen et al., 2013, 2016).

**Figure 5:**
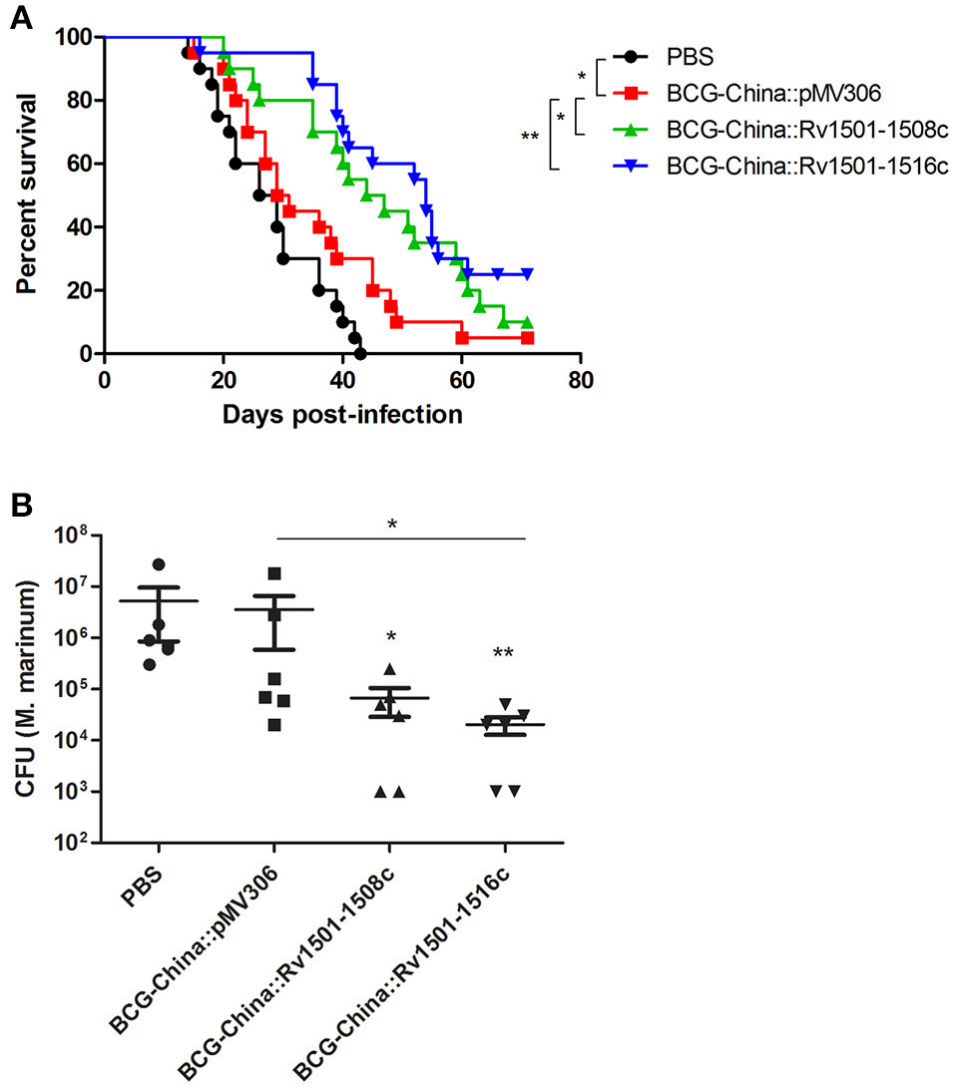
Protective efficacy of RD4 knock-in *M. bovis* BCG. **(A)** Survival curves of zebrafish vaccinated with recombinant BCG-China and challenged with *M. marinum* 535. Groups of adult zebrafish (*n* = 20) were vaccinated with 10^4^ CFU of BCG strains or PBS. Thirty days after BCG vaccination, zebrafish were infected with 10 CFU of *M. marinum* 535 and were monitored for survival. Log-rank analysis was performed for statistical significance. ^*^p < 0.05; ^**^p < 0.01. **(B)** Bacterial burdens of zebrafish vaccinated with recombinant BCG-China and challenged with *M. marinum* 535. Groups of zebrafish (*n* = 15) were vaccinated with 10^4^ CFU of BCG strains or PBS. Thirty days after BCG vaccination, zebrafish were infected with 10 CFU of *M. marinum* 535. After 30 days of *M. marinum* infection, six live fish from each group were sacrificed and the number of *M. marinum* in each fish was determined. Kruskal-Wallis test followed by Dunn's Multiple Comparison test was performed. (^*^p < 0.05; ^**^p < 0.01.). The *M. marinum* burden between the BCG-China::Rv1501-1516c and the parental BCG groups was statistically significant (*p* < 0.05). The *M. marinum* burden in both recombinant BCG groups was also significantly lower than that in the PBS group.

Groups of adult zebrafish (*n* = 20) were vaccinated intraperitoneally with 10^4^ CFU of BCG strains or PBS as the negative control. Thirty days after BCG vaccination, zebrafish were infected intraperitoneally with 10 CFU of *M. marinum* 535 and were monitored for survival. Log-rank analysis of the survival curves revealed that the median survival time of the unvaccinated group (PBS), the parental BCG, the BCG-China::Rv1501-1508c and BCG-China::Rv1501-1516c groups were 27.5, 30, 45.5, and 54 days, respectively (Figure 5A). Zebrafish vaccinated with BCG-China::Rv1501-1508c and BCG-China::Rv1501-1516c both survived significantly longer than those vaccinated with the parental BCG. The BCG-China::Rv1501-1516c group appeared to have the best protection, although the difference between the two BCG knock-in strains was not statistically different. As a control, the difference between the BCG-China and the PBS groups was statistically significant (*p* = 0.03).

In a separate experiment, groups of zebrafish (*n* = 15) were vaccinated by the BCG strains and infected with *M. marinum* as described above, and six live fish from each group were sacrificed at 30 days post-infection to determine the bacterial burdens. Consistent with the survival curve data, the *M. marinum* burdens in the BCG-China::Rv1501-1508c and BCG-China::Rv1501-1516c groups were 1.73 and 2.25 log_10_ lower than that in the BCG-China group, respectively (Figure 5B). The difference between the BCG-China::Rv1501-1516c and the parental BCG groups was statistically significant (*p* < 0.05) and the bacterial burdens in the two knock-in BCG groups were also significantly lower than the unvaccinated group (Figure 5B).

## Discussion

The RD4 of *M. tb* H37Rv is present in most members of the MTBC including all the examined strains of *M. tb, M. africanum, M. canettii*, and *M. microti* (Brosch et al., 2002). However, it is absent in the most common and classical *M. bovis* strains, which were isolated from cattle from Argentina, the Netherlands, the UK, and Spain, as well as from humans (van Soolingen et al., 1994; Brosch et al., 2002). In contrast, *M. bovis* strains isolated from oryx, seal, and goat contain RD4 (Brosch et al., 2002), giving rise to speculation that RD4 may play a role in host interactions. As the first attempt to address this question, our current study examined the potential role of RD4 in virulence of *M. bovis* BCG and *M. marinum*. All BCG strains, derived from *M. bovis*, lack RD4 (Mahairas et al., 1996; Brosch et al., 1998, 2002; Behr et al., 1999; Gordon et al., 1999; Mostowy et al., 2003) and thus they represent a convenient model to address the role of RD4 in *M. bovis* virulence. On the other hand, *M. marinum* contains an extended RD4 which include homolgs of Rv1502, Rv1504c to Rv1508c (Ren et al., 2007), therefore allowing us to examine if the presence of an extra copy of these genes affects virulence of *M. marinum*. Our results showed that knock in of a fragment containing the entire RD4 (Rv1501-1516c) in *M. marinum* increased its virulence, whereas this effect was not observed in *M. bovis* BCG (Figure 4). It is possible that RD4 works in synergy with other virulence factors present in *M. marinum* but not in BCG. Alternatively, since BCG is already highly attenuated, introducing the RD4 alone may not provide a noticeable effect on its virulence. Future studies to use *M. bovis* that lacks RD4 and construct RD4 knock-in *M. bovis* strain may help to address this question. Similarly, construction and evaluation of an RD4 deletion strain of *M. marinum* will help to confirm its role in virulence.

Interestingly, addition of the partial RD4 (Rv1501-1508c) in BCG and *M. marinum* appeared to decrease the virulence of these strains (Figure 4). The function of most proteins encoded in RD4 remains unknown but they appear to be involved in the biosynthesis of trehalose containing glycolipids. Many genes at the extended RD4 locus in *M. marinum* are involved in the biosynthesis of glycosylated acyltrehalose LOSs (Burguiere et al., 2005; Ren et al., 2007; van der Woude et al., 2012). Rv1511 and Rv1512 are predicted nucleotide-sugar dehydratase and epimerase, respectively. Rv1516c is a probable sugar transferase. MMAR_2327, homolog of Rv1508c, was shown to be involved in the biosynthesis of LOSs in *M. marinum* (van der Woude et al., 2012). Although *M. tb* H37Rv does not synthesize LOSs, transposon inactivated mutants of Rv1503c and Rv1506c were impaired in the synthesis of other aceyltrehalose containing lipids, namely 2,3-di-*O*-aceyltrehalose (Brodin et al., 2010). The Rv1503c and Rv1506c mutants of *M. tb* also failed to induce phagosome maturation arrest in infected macrophages and were attenuated in virulence (Brodin et al., 2010). In contrast, disruption of genes in the biosynthetic locus of LOSs in *M. marinum* resulted in increased virulence in zebrafish embryo infection model (van der Woude et al., 2012). These findings, together with data from our current study, suggest a variable role of RD4 in mycobacterial virulence, apparently in a species-specific manner, and may reflect the complex interactions between the host and the pathogen.

One significant finding from our studies is that while RD4 knock-in did not significantly affect the virulence of BCG, it did improve its protective efficacy (Figure 5). In *M. marinum*, the biosynthesis of LOSs was found to be linked to the secretion of PE-PGRS proteins (van der Woude et al., 2012). Disruption of the full-length LOS molecules was found to reduce the release of PE-PGRS proteins from the *M. marinum* cell surface (van der Woude et al., 2012). PE-PGRS are members of the PE/PPE family proteins, and many PE/PPE proteins have been shown to modulate host immune responses (Mukhopadhyay and Balaji, 2011; Sampson, 2011) and play a critical role in mycobacterial pathogenesis (Fishbein et al., 2015). Introduction of RD4 into BCG may alter its surface properties and facilitate the release of PE-PGRS proteins, thereby improving antigen presentation and protection.

The lack of RD4 in BCG strains may also be a disadvantage in terms of providing protection against *M. tb* since all strains of *M. tb* contains RD4. Future studies to evaluate the protective efficacy the RD4 knock-in BCG against *M. tb* and *M. bovis* (including strains with or without RD4) in animal models will help to address this question. While further studies will be required to test our hypotheses, the RD4 knock-in BCG could be a promising candidate for the development of more effective TB vaccines to replace current BCG.

## Author contributions

JL conceived and supervised the project, wrote the paper. LZ supervised the project. HR, XL, CL, JY, FC, and RS performed the experiments.

### Conflict of interest statement

The authors declare that the research was conducted in the absence of any commercial or financial relationships that could be construed as a potential conflict of interest.
